# Differences and Mechanism of Waxy Corn Starch and Normal Corn Starch in the Preparation of Recrystallized Resistant Starch (RS3)

**DOI:** 10.3390/foods13132039

**Published:** 2024-06-27

**Authors:** Qing Su, Lirong Chen, Linlin Sun, Kaichang Liu, Kuijie Gong

**Affiliations:** 1 Crop Research Institute, Shandong Academy of Agricultural Sciences, North Industrial Road 202, Jinan 250100, China; suqingzws@163.com (Q.S.); 13047481469@163.com (L.C.); linlins4355@yeah.net (L.S.); 2 Shandong Academy of Agricultural Sciences, North Industrial Road 202, Jinan 250100, China; liukc1971@163.com

**Keywords:** waxy corn starch, normal corn starch, resistant starch content, influencing mechanism

## Abstract

This study prepared resistant starch (RS) from waxy corn starch and normal corn starch and analyzed the effects of its molecular and microstructural characteristics on RS content. The RS content of waxy corn resistant starch (RS-WCS) was highest at 57.8%, whereas that of normal corn resistant starch (RS-NCS) was 41.46%. The short-chain amylose contents of RS-WCS and RS-NCS were 47.08% and 37.24%, respectively, proportional to their RS content. Additionally, RS content positively correlated with crystallinity, short-range order degree, and degree of polymerization (DP), exceeding 25. Electron microscopic images, before and after enzymolysis, revealed that RS-WCS was hydrolyzed from the surface to the center by pancreatic α-amylase, while RS-NCS underwent simultaneous hydrolysis at the surface and center. These results indicate that the higher RS content in RS-WCS, compared to RS-NCS, is attributable to the synergistic effects of molecular structure and microstructure.

## 1. Introduction

Resistant starch (RS) is an enzyme-resistant starch that remains undigested and unabsorbed in the small intestine but can be fermented into short-chain fatty acids by colon microorganisms [1]. It is often added to foods as a food additive, providing benefits such as inhibiting postprandial blood glucose spikes, reducing blood lipids, and fostering healthy intestinal bacterial growth [2,3]. This type of starch, termed recrystallized starch when prepared through the decomposition of starch into α-glucan short chains followed by controlled crystallization [4], has its content influenced by the amylose and amylopectin levels in the raw starch [5]. In addition, crystal type, crystallinity, branched-chain molecular weight distribution, double-helical structures, and microstructure were found to be important factors affecting the RS content [6].

Amylose plays an important role in RS content; Iacovino et al. [7] established that RS content is directly proportional to amylose content. For instance, Reddappa et al. [8] observed that corn starch with amylose content ranging from 1.7% to 66.2% exhibited RS levels between 1.4% and 39.4%. Similarly, the RS content in soft rice starch correlates with amylose levels [9]. High-amylose corn starch (70% amylose content) can yield RS content up to 43.4% [10]. Fang et al. [11] prepared resistant starch using waxy and normal potato starches and noted that digestibility was positively correlated with natural amylose content. However, Kiatponglarp et al. [12] argued that a lack of amylose does not impact RS content, demonstrating that RS3 prepared from waxy rice starch could reach as high as 74.5%. The variation in findings may be attributed to an overlooked importance of short-chain amylose crystals.

The factors influencing the content of resistant starch are a subject of debate. In the process of preparation of resistant starch, higher concentrations of solids, elevated temperatures, and shorter chain lengths favor the formation of A-type crystals, whereas the opposite conditions facilitate the formation of B-type crystals. A-type crystals exhibit greater resistance to digestion than B-type crystals [4]. The higher enzyme resistance of the aggregates of A-type crystallites was due to the dense and compact morphology with reduced accessibility of double helices to the enzymes. RS3, recrystallized from long-chain alpha-glucan, is more indigestible than its short-chain counterparts. However, Klostermann et al. [13] contend that crystal type has a greater influence on RS content than chain length. Starches within the crystallinity region demonstrate higher resistance to digestibility, but overlooking the digestibility of V-type recrystallization in the non-crystalline region could lead to a non-correlation between RS content and relative crystallinity [14]. The impact of starch branched chain length distribution on RS content is significant. RS prepared from waxy rice starch, which has relatively low dispersion, exhibits a 10% higher content than natural rice starch with polydispersion [15]. To date, the role of microstructure in starch resistance to enzymatic hydrolysis has not been adequately emphasized. It is suggested that the surface of RS3 is relatively dense, requiring digestive enzymes to initiate hydrolysis from the surface [16]. The particles of waxy corn RS3 are in a state of spherulite accumulation [17], whereas those of high straight-chain RS3 appear as irregular gel blocks [18]. Therefore, studying the hydrolysis of RS with varying particle morphologies and properties is essential.

In this study, high RS was prepared from waxy corn starch and normal corn starch. By analyzing the amylose content, crystallites, short-range molecular orders, double helicity, amylopectin chain length distribution, and microscopic morphology of the two starches, the significant influence of these variables on RS content was delineated, and the hydrolysis mechanisms were explored from a microstructural perspective. These findings offer valuable insights for the preparation of resistant starch.

## 2. Materials and Methods

### 2.1. Materials

Waxy corn starch (WCS) and normal corn starch (NCS) were sourced from Foshan Guonong Starch Co., Ltd. (Foshan, China). Pululanase (LP482001, ≥2000 U/mL) was obtained from Cool Chemical science and technology Co. (Beijing, China). Isoamylase (ZME-E-ISAMY, 200 U/mL), resistant starch test kit (K-RSTAR, 100 Assays per Kit), and the amylose test kit (K-AMYL, 100 Assays per Kit) were acquired from Megazyme International Ireland Ltd. (Wicklow, Ireland). All other chemical reagents were of analytical grade.

### 2.2. Preparation of Resistant Starch (RS)

A starch suspension was prepared using a 1:10 starch-to-water ratio. The pH of the emulsion was adjusted to 4.5 with 0.1 mol/L citric acid. This mixture was pre-gelatinized using a microwave before being subjected to high temperature and pressure (121 °C) for 20 min to ensure complete gelatinization. Upon removal, the mixture was cooled to 60 °C, and pullulanase was added at a dosage of 5 U/g. Following enzymatic reaction for 3 h, the enzyme was deactivated in water bath at 85 °C for 10 min, cooled to 50 °C, and isoamylase was added at 1 U/g. The mixture was then oscillated in thermostatic oscillator at 50 °C for 24 h; the isoamylase was deactivated in a water bath at 85 °C for 10 min. After completion, the product was stored at 4 °C for 24 h to recrystallize the recovered starch, then dried at 45–55 °C, ground into powder, and stored in a freezer at −18 °C.

### 2.3. Determination of RS Content

Resistant starch content was measured using the Megazyme resistant starch kit (K-RSTAR, Megazyme International Ireland Ltd., Bray, Ireland). The process involved mixing the sample with pancreatic α-amylase (10 mg/mL) and amyloglucosidase (3 U/mL) and incubating on a shaking table at 37 °C with continuous agitation (200 rpm) for 16 h. The reaction was halted by adding ethanol, and the mixture was washed twice with alcohol. The precipitate was then dissolved in 2 mol/L KOH. After adjusting the pH, glucose was liberated from the sample by amyloglucosidase (3300 U/mL). The absorbance at 510 nm was measured using GOPOD as chromogenic agent and the resistant starch content was quantified based on the glucose content. Resistant starch content (%) was calculated on a dry weight basis for test samples according to AOAC 2002.02 [19].

### 2.4. Determination of Amylose Content in RS

Amylose content was determined using the amylose assay kit (K-AMYL, Megazyme). The method involves concanavalin A (Con A) forming complexes with branched polymers, which are precipitated, whereas linear amylose remains in solution. The concentration of amylose is estimated by comparing the absorbance at 510 nm of the supernatant (from the Con A-precipitated sample) to that of the total starch sample [20].

### 2.5. Determination of Moisture Content in RS

Moisture content was determined according to the GB 5009.3-2016 standard. A clean, flat aluminum weighing bottle was dried to constant weight, recorded as m_1_. Then, 3 g of the sample, measured accurately to 0.0001 g, was added to the weighing bottle, with the combined mass recorded as m_2_. The sample was dried in an oven at 101–105 °C for 3 h, repeated twice, with a maximum weighing error of 2 mg. The final mass of the sample and weighing bottle was recorded as m_3_. The moisture content was calculated using the following formula:

Moisture content% = (m_2_ − m_3_)/(m_2_ − m_1_) × 100 (1)

### 2.6. Differential Scanning Calorimetry Analysis of RS (DSC)

Differential scanning calorimetry (DSC) was performed using a DSC 204F1 (NETZSCH, Bavaria, Germany). Starch samples (3 mg) were placed into hermetic aluminum pans, with 6 μL of deionized water added. The pans were hermetically sealed and equilibrated overnight at room temperature. DSC runs were conducted from 30 to 140 °C at a heating rate of 10 °C/min [12].

### 2.7. Fourier Transform Infrared Spectroscopy of RS (FTIR)

Starch samples (1.00 mg) were thoroughly mixed with KBr (150 mg) and pressed into a transparent tablet. FTIR spectra were recorded using a Nicolet-iS20 FTIR spectrometers (Thermo Fisher Scientific Inc., Waltham, MA, USA) over a spectral range of 4000–400 cm^−1^, at a resolution of 4 cm^−1^.

### 2.8. X-ray Diffraction of RS (XRD)

Samples were equilibrated in a sealed dryer with a saturated potassium chloride solution for 12 h prior to analysis. XRD was performed using an XRD D-MAX 2500 (Rigaku Corporation, Tokyo, Japan), employing a Cu-Kα radiation source (40 kV, 30 mA). Scanning conditions were as follows: 2θ angle range of 3–40°, step size of 0.02°, and scanning rate of 6°/min.

### 2.9. Determination of Amylopectin Chain Length Distribution in RS

Following the method of Klostermann et al. [13], approximately 10 mg of starch was suspended in 5 mL of water and subjected to a boiling water bath for 60 min, with intermittent vortex mixing. Subsequently, 50 μL of sodium acetate (0.6 mol/L, pH 4.4), 10 μL of NaN_3_ (2% *w*/*v*), and 10 μL of isoamylase (1400 U) were added, and the mixture was incubated at 37 °C for 24 h. A 0.5% (*w*/*v*) sodium borohydride solution was added, mixed vigorously, and left for 20 h. The mixture was then transferred to a centrifuge tube, dried under nitrogen at room temperature, dissolved in 30 μL of 1 M NaOH for 60 min, diluted with 570 μL of water, and centrifuged at 12,000 rpm for 5 min. The supernatant was collected for analysis.

Chromatographic analysis was conducted using a Thermo ICS5000 ion chromatography system (Thermo Fisher Scientific, Waltham, MA, USA) equipped with an electrochemical detector. The Dionex™ CarboPac™ PA200 liquid chromatography column (250 × 4.0 mm, 10 μm) was used with an injection volume of 5 μL. The mobile phases consisted of 0.2 M NaOH (A phase) and a mixture of 0.2 mol/L NaOH/0.2 mol/L NaAC (B phase), with a column temperature of 30 °C. The elution gradient was set as follows: 0–10 min, 90:10 (A/B, *v*/*v*); 30–50 min, 40:60 (A/B, *v*/*v*); 50.1–60 min, 90:10 (A/B, *v*/*v*), at a flow rate of 0.4 mL/min.

### 2.10. Observation by Scanning Electron Microscope (SEM)

Starch samples were evenly dispersed on conductive adhesive, coated with gold, and examined using an Apreo SEM (Thermo Fisher Scientific, Waltham, MA, USA) at an acceleration voltage of 20 kV and a magnification of 5000×.

### 2.11. Statistical Analysis

Origin 2022 was used for graphing and Pearson’s correlation analysis. The crystallinity derived from XRD data was calculated using MDI Jade 6.0 software. Statistical analyses, including mean values and standard deviations, were conducted using SPSS version 21.0, followed by Duncan’s multiple range test. The significance level was set at *p* < 0.05.

## 3. Results

### 3.1. Differences in RS Content and Moisture Content of the Two RS Type

As indicated in Table 1, the RS content of waxy corn resistant starch (RS-WCS) and normal corn resistant starch (RS-NCS) was significantly higher than that of WCS and NCS (*p* < 0.05), with RS-WCS having the highest RS content at 57.80%. The moisture content of RS was lower than that of the original starches.

### 3.2. Differences in Thermal Properties of the Two RS Type

As indicated in Table 2, the gelatinization temperatures of native starches are consistently close. The onset temperature (T_o_) for RS-WCS is 100.40 °C, and for RS-NCS, 98.77 °C; these values are significantly higher than those of their corresponding raw starches. Similarly, the peak temperature (T_p_) and conclusion temperature (T_c_) exhibit analogous trends, supporting the findings of Shrestha et al. [21]. The enthalpy change (ΔH) for RS-WCS and RS-NCS was found to be greater than that for WCS and NCS, suggesting a positive correlation with the content, organization, and stability of the double helix structure [22]. These results indicate that the RS3 prepared possesses enhanced thermal stability.

### 3.3. Influence of Amylose Content on RS Content

**Figure 1:**
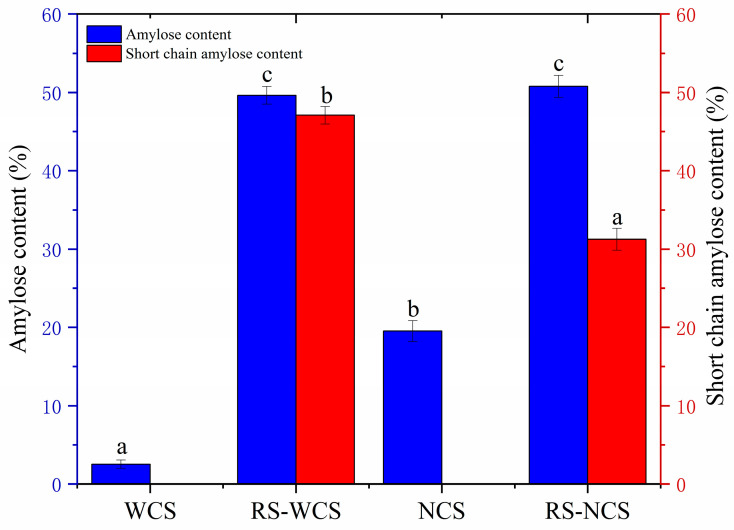
Change of amylose content in RS. The values are expressed as the mean of three parameters ± standard deviation. Different letters in a column signify significant differences (*p* < 0.05).

As depicted in Figure 1, the amylose content of NCS is higher than that of WCS, but the amylose content of RS-WCS (49.63%) is nearly identical to that of RS-NCS (50.78%). According to the mechanism where Con A forms complexes with branched polymers and precipitates [20], the detected amylose content includes both short-chain amylose and natural amylose. The natural amylose in RS-WCS and RS-NCS corresponds to the amylose in WCS and NCS, allowing for the calculation that the short-chain content in RS-WCS is 47.08% and in RS-NCS is 31.24% (Figure 1). Pearson’s correlation analysis indicates a correlation coefficient of 0.93 between amylose content and RS content, though the correlation is not statistically significant (*p* > 0.05). While the natural linear chain content has minimal impact on RS content, the short-chain content is directly proportional to RS content, aligning with findings by Kiatponglarp et al. [12]. Short straight-chain recrystallized starch can form a more perfect crystal.

### 3.4. Differences in Chemical Structure of RS

**Figure 2:**
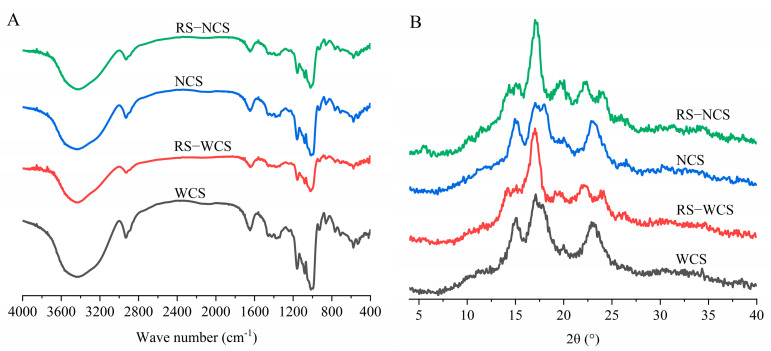
FTIR spectra (**A**) and XRD spectra (**B**) of RS.

FTIR spectra can be divided into four regions: below 800 cm^−1^, 800–1500 cm^−1^(fingerprint region), 2800–3000 cm^−1^ (C-H stretching region), and 3000–3600 cm^−1^ (O-H stretching region) [23]. As shown in Figure 2A, the fundamental IR spectra profiles of the four starch types are similar, indicating that no new functional groups were formed during preparation, and their chemical structure remained unchanged, involving only the breakage and recombination of hydrogen bonds between starch chains.

The ratios of 1047/1022 and 995/1022, calculated from infrared results and presented in Table 3, indicate the proportion of ordered to amorphous structure (degree of order) and double helicity, respectively. The double helicity of the four starches is higher than their degree of order. Tang et al. [18] posited that the higher double helicity compared to the degree of order is due to starch chains forming linear single/double helices, which do not fully engage in the ordered structure arrangement within the crystal region. Enhanced hydrolysis resistance led to significantly increased degree of order and decreased double helicity in RS-WCS and RS-NCS, suggesting that short-chain double-helix rearrangement after resistant starch formation increases the degree of order.

The degree of order for RS-WCS and RS-NCS are higher than those for WCS and NCS, respectively, with RS-WCS exhibiting the highest degree of order, though no significant difference exists between RS-NCS and RS-WCS. Compared to ordinary corn starch, RS treatment of waxy corn starch markedly enhances the original starch’s degree of order. Guo et al. [24] noted that amylose inhibits double helix rearrangement in starch, leading to a reduction in order degree. Pearson’s correlation analysis revealed a correlation coefficient of 0.75 between order degree and RS content, but no significant correlation exists (*p* > 0.05), indicating that order degree is not a decisive factor for RS content.

### 3.5. Differences in Crystal Structure and Crystallinity between the Two RS Types

X-ray diffraction patterns reveal the characteristic patterns of different crystalline starches. As shown in Figure 2B, WCS and NCS exhibit peaks at 2θ values of 15.04°, 17.01°, 17.83°, and 23.06°, forming an A-type structure; RS-WCS peaks at 2θ values of 14.18°, 17.01°, 22.10°, and 24.12°, indicating a B-type structure; RS-NCS shows peaks at 2θ values of 5.54°, 14.85°, 17.01°, 19.60°, 22.24°, and 23.88°, corresponding to a B + V structure, aligning with findings by Miao et al. [25]. Starch can be converted to resistant starch type 3 (RS3) through gelatinization and recrystallization treatments. Following low-temperature (4 °C) treatment, the recrystallization of RS3 predominantly exhibits a B-type structure [4].

As indicated in Table 3, the crystallinity of resistant starches increased compared to that of raw starches. Specifically, the crystallinity of RS-WCS reached the highest at 42.88%, and the crystallinity of RS-NCS increased from 23.24% in NCS to 27.03%. Kiatponglarp et al. [12] suggest that this increase in crystallinity results from the formation of more perfectly structured and densely recrystallized starch due to moist heat treatment. The crystallinity of RS-WCS rose from 36.38% in WCS to 42.31%, while the RS content surged from 0.36% in WCS to 57.80%. This indicates that the rise in RS content may not solely be attributed to increased crystallinity, a finding also reported by Ding et al. [14].

### 3.6. Changes in Branched Chain Length Distribution of Amylopectin between the Two RS Types

**Figure 3:**
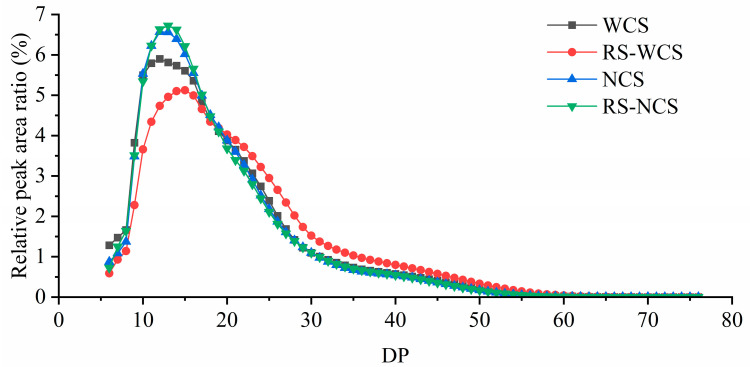
Distribution of amylopectin chain length of RS.

As illustrated in Figure 3, the degree of polymerization (DP) distribution of RS-WCS shifted rightward compared to WCS, indicating an increase in the content of high DP molecules. The average DP of RS-WCS (21.63) was significantly higher than that of WCS (19.32) (*p* < 0.05), whereas the DP distribution of RS-NCS remained essentially unchanged from NCS, with an average DP of approximately 18.90. According to Klostermann et al. [13], high DP is more resistant to digestion than lower DP.

### 3.7. Changes in Branched Chain Length Distribution of Amylopectin between the Two RS Types

As illustrated in Figure 3, the degree of polymerization (DP) distribution of RS-WCS shifted rightward compared to WCS, indicating an increase in the content of high-DP molecules. The average DP of RS-WCS (21.63) was significantly higher than that of WCS (19.32) (*p* < 0.05), whereas the DP distribution of RS-NCS remained essentially unchanged from NCS, with an average DP of approximately 18.90. NCS may induce short-chain double-helices to form an amorphous structure due to high natural amylose content [23], inhibiting the precipitation of the A-chain, thus maintaining a similar amylopectin chain length distribution between RS-NCS and NCS. According to Klostermann et al. [13], high DP is more resistant to digestion than lower DP.

The amylopectin structure is categorized into short chain (DP < 6), A-chain (DP 6–12), B1-chain (DP 13–24), B2-chain (DP 25–36), and B3-chain (DP > 36) [6]. Quantitative results from DP 6–76, displayed in Table 2, show that the B1-chain is the predominant fraction in each sample group. The content of A-chain (DP 6–12) and B1-chain (DP 13–24) in RS-WCS is lower than in WCS, but the content of B2-chain (DP 25–36) and B3-chain (DP > 36) is higher. The amylopectin structure of RS-NCS is similar to that of NCS. According to Villas-Boas et al. [22], short chains (A-chain) struggle to form double-helices and may disrupt the organization of the crystalline structure, causing defects in the crystalline layers of particles. Conversely, a high proportion of medium chain (B1) increases steric hindrance, which facilitates regeneration. Longer chains (B2 and B3) form more double-helices, enlarging the crystal space volume and decreasing hydrolysis efficiency. WCS can reduce the proportion of A-chain and increase that of B2 and B3 through RS3 treatment. In contrast, the chain length distributions of RS-NCS and NCS were almost identical. Correlation analysis between DP > 25 content and RS content yielded a correlation coefficient of 0.69 (*p* > 0.05), indicating no significant correlation. Therefore, chain length distribution was not the primary factor driving the differential increase in resistance between RS-WCS and RS-NCS.

### 3.8. Microstructure Changes of RS

**Figure 4:**
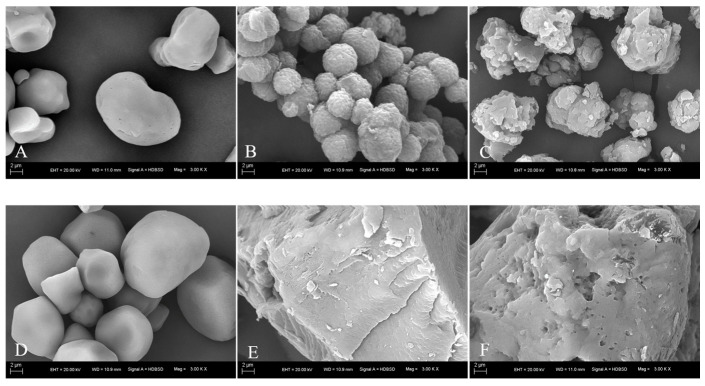
SEM of RS. WCS (**A**), RS-WCS (**B**), RS-WCS after enzymatic hydrolysis (**C**), NCS (**D**), RS-NCS (**E**), and RS-NCS after enzymatic hydrolysis (**F**).

As depicted in Figure 4A–C, WCS exhibits an irregular shape with micropores on its surface. RS-WCS is characterized by a state of spherulite aggregation with a rough surface where microscopic lamellar structures are observable. After enzymatic hydrolysis, large layers remain on the surface of the particles, with the aggregation sites remaining largely intact. Shi et al. [17] suggest that RS-WCS consists of small spherulites formed by the radial arrangement of short-chain double-helix crystal sheets, interconnected by larger crystal sheets, which stabilize the aggregation of RS-WCS particles. Das et al. [26] observed through clinical microscopy that recrystallized starch displays a denser crystalline layer than the original starch. Furthermore, the interaction among the aggregated small spherical crystals impedes water absorption and expansion of the particles [16]. Consequently, amylase can only hydrolyze the surface of the particles, contributing to the increased RS content in RS-WCS.

As shown in Figure 4D–F, the particle morphology of NCS closely resembles that of WCS. RS-NCS does not exhibit a separate particle state but aggregates into a gel-like form with a lamellated surface structure and sharp edges. Post-enzymatic hydrolysis, RS-NCS particles become looser, displaying hydrolytic channels on the surface and large residual layers with rounded edges. Zeng et al. [27] formulated RS by mixing amylose and amylopectin in ratios of 1:1 or 1:2, resulting in the formation of gel chunks. Thus, a high content of natural amylose during the regenerative process prevents recrystallization into spherical particles. Analogous to RS-WCS, in RS-NCS, the short, branched double-helix predominates in the crystalline lamellae [18], while long straight chains are interspersed between lamellae [28]. Amylase penetration into RS-NCS particles loosens them, which directly results in higher RS content in RS-WCS compared to RS-NCS.

**Figure 5:**
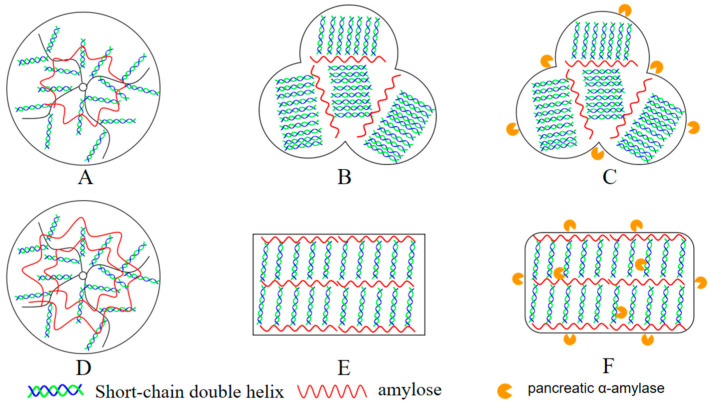
Schematic of the hydrolysis reaction of two RSs. WCS (**A**), RS-WCS (**B**), RS-WCS after enzymatic hydrolysis (**C**), NCS (**D**), RS-NCS (**E**), and RS-NCS after enzymatic hydrolysis (**F**).

The hydrolysis process of two resistant starches is schematically depicted in Figure 5.

## 4. Conclusions

This study primarily focused on analyzing the amylose content, short-range order degree, crystallinity, molecular weight distribution, and microstructure of RS-WCS and RS-NCs, as well as their impacts on RS3 content. Results indicate that the RS content of RS-WCS is higher than that of RS-NCS. Both RS-WCS and RS-NCS have an amylose content of approximately 50%, with thorough enzymatic hydrolysis, and RS content is proportional to the content of short-chain amylose. The chemical structures of RS-WCS and RS-NCS remained unchanged, with RS-WCS displaying a B-type and RS-NCS a B + V-type structure. The IR1047/1022 (0.934) and crystallinity (42.31%) of RS-WCS are higher than those of RS-NCS. Additionally, the content of B2 (DP 25–36) and B3 (DP 36–76) chains in RS-WCS is greater than in RS-NCS. Correlation analysis revealed that RS content is positively correlated with amylose content, IR1047/1022, and B2 + B3 content, although these correlations are not significant. This correlation offers a method for identifying and screening RS3s with varying RS contents. SEM analysis shows that RS-WCS particles are small spherulite aggregates, undergoing hydrolysis from the surface inward, while RS-NCS particles are gel blocks that simultaneously hydrolyze internally and on the surface due to the presence of hydrolysis channels.

In conclusion, the synergistic effects of short-chain amylopectin content, order degree, crystallinity, long-chain content (DP > 25), and microstructure on RS-WCS and RS-NCS provide a theoretical foundation for the development of resistant starches with elevated RS content.

## Figures and Tables

**Table 1:** RS content and moisture content of RS.

Sample	RS Content (%)	Moisture Content (%)
WCS	0.36 ± 0.08 ^a^	11.48 ± 0.03 ^c^
RS-WCS	57.80 ± 0.91 ^c^	9.99 ± 0.18 ^b^
NCS	1.06 ± 0.10 ^a^	12.80 ± 0.08 ^d^
RS-NCS	41.46 ± 0.87 ^b^	7.41 ± 0.03 ^a^

The values for RS content and moisture content are reported as the mean ± standard deviation of three measurements. Values in the same column followed by different superscripts are significantly different (*p* < 0.05). Samples were stored at −18 °C prior to analysis.

**Table 2:** Thermal properties of RS.

Sample	T_o_ (°C)	T_p_ (°C)	T_c_ (°C)	ΔH (J/g)
WCS	61.40 ± 1.15 ^a^	69.13 ± 0.40 ^a^	74.17 ± 1.55 ^a^	4.29 ± 0.28 ^a^
RS-WCS	100.40 ± 2.13 ^c^	102.97 ± 1.62 ^b^	113.50 ± 1.10 ^b^	16.79 ± 0.29 ^c^
NCS	65.67 ± 1.15 ^b^	71.07 ± 1.06 ^a^	75.77 ± 0.72 ^a^	4.58 ± 0.31 ^a^
RS-NCS	98.77 ± 1.30 ^c^	101.00 ± 1.41 ^b^	112.30 ± 0.70 ^b^	15.01 ± 0.58 ^b^

The values for T_o_, T_p_ T_c_, and ΔH are reported as the mean ± standard deviation based on three measurements. Values within the same column followed by different superscripts indicate significant differences (*p* < 0.05). T_o_, T_p_, and T_c_ = onset, peak, and conclusion temperatures, respectively; ΔH = enthalpy change. Samples were stored at −18 °C before analysis.

**Table 3:** Distribution of amylopectin chain length, IR ratio, and relative crystallinity of RS.

Sample	DP 6–12	DP 13–24	DP 25–36	DP36–76	IR1047/1022	IR995/1022	Relative Crystallinity %
WCS	25.43	52.55	14.82	7.20	0.866 ± 0.009 ^a^	0.995 ± 0.006 ^a^	36.38 ± 0.887 ^b^
RS-WCS	17.68	51.72	20.14	10.46	0.934 ± 0.006 ^bc^	0.952 ± 0.010 ^b^	42.31 ± 1.28 ^d^
NCS	25.15	54.43	14.08	6.34	0.914 ± 0.008 ^b^	0.994 ± 0.003 ^a^	22.81 ± 1.43 ^a^
RS-NCS	25.33	54.25	14.05	6.37	0.930 ± 0.005 ^c^	0.950 ± 0.001 ^b^	27.23 ± 0.99 ^b^

The values for IR1047/1022, IR995/1022, and relative crystallinity are reported as the mean ± standard deviation of three measurements. Values in the same column followed by different superscripts are significantly different (*p* < 0.05).

## Data Availability

The original contributions presented in the study are included in the article, further inquiries can be directed to the corresponding author.
